# TB Peritonitis Mistaken for Ovarian Carcinomatosis Based on an Elevated CA-125

**DOI:** 10.1155/2012/215293

**Published:** 2012-02-20

**Authors:** Joseph D. Boss, Christopher T. Shah, Oladoyin Oluwole, John N. Sheagren

**Affiliations:** ^1^College of Human Medicine Michigan State University, East Lansing, MI 48824, USA; ^2^Department of Internal Medicine, Grand Rapids Medical Education Partners, Grand Rapids, MI 49503, USA

## Abstract

*Background*. In the United States, tuberculosis (TB) is of relatively low prevalence and most newly diagnosed patients are born outside of the United States. In addition, a large percentage (20.6%) of TB cases initially present with extrapulmonary manifestations (CDC, 2010). Cases of TB peritonitis are a diagnostic challenge in women due to the nonspecific clinical features overlapping with signs of ovarian cancer. (Kosseifi et al., 2009; Rashed et al., 2007; and Xi et al., 2010). We present a 27 year-old woman thought to have ovarian carcinomatosis based on elevated levels of CA-125 who was ultimately diagnosed with TB salpingitis, endometritis, and peritonitis. *Methods*. This brief report is a retrospective case report. *Results*. This case outlines the unfortunate consequences of the misdiagnosis of what probably was an antibiotic responsive illness, resulting in an unnecessarily aggressive surgical procedure. The delay in the diagnosis of tuberculous pertitonitis resulted in an unnecessary radical resection of the patient's reproductive organs. *Conclusions*. Patients with TB peritonitis present with non-specific signs that may be misdiagnoses as ovarian cancer. In differentiating between ovarian carcinomatosis and peritoneal TB, it is vital to consider country of origin, age, CA-125, ascitic fluid analysis, and the use of intra-operative frozen sections.

## 1. Background

In the United States, tuberculosis (TB) is of relatively low prevalence, and most newly diagnosed patients are born outside of the United States. In addition, a large percentage (20.6%) of TB cases initially present with extrapulmonary manifestations [1]. These cases of TB pose a diagnostic challenge, especially rare presentations such as TB peritonitis [2]. Delay in diagnosis of peritoneal TB can often be more than four months due to the nonspecific clinical features such as abdominal distension, ascites, abdominal tenderness, fever, and weight loss [2, 3]. Further complicating the clinical picture, elevated levels of CA-125, which has a low specificity in ovarian peritoneal carcinomatosis, may be elevated in peritoneal TB and lead to the erroneous diagnosis of ovarian cancer [3–5]. Given the need for extensive debulking in ovarian carcinoma, women with peritoneal TB may be unnecessarily subjected to extended surgery including the removal of reproductive organs [4]. We present a 27-year-old woman thought to have ovarian carcinomatosis based on elevated levels of CA-125 who was ultimately diagnosed with TB salpingitis, endometritis, and peritonitis.

## 2. Case Summary

The patient is a 27-year-old woman who emigrated from Guatemala ten years prior to presentation with no significant past medical history. She was not aware of ever having a tuberculosis skin test or of any prior exposure to tuberculosis. Eight months earlier, she had undergone her third normal spontaneous vaginal delivery. Two months prior to presentation, the patient began to experience abdominal pain and distention. At the time of presentation, abdominal ultrasound demonstrated ascites and bilateral adnexal masses. CT scan suggested tubular structures within the adnexa, demonstrating circumferential enhancement. Initial paracentesis revealed a white cell count of 1330 (78% lymphocytes) and serum-ascites albumin gradient of 0.2 g/dL. All cultures and acid-fast bacillus smears were negative at that time. Recurrent ascites prompted repeat paracentesis which yielded 1300 mL of greenish fluid that was again culture and acid-fast bacillus smear negative. Two days following the initial paracentesis, the patient was readmitted for persistent abdominal distention and pain, fevers, nausea, vomiting, chills, and night sweats. Repeat CT scan showed pronounced thickening of the peritoneum, a large amount of ascites, and dilation of the fallopian tubes. Serum CA-125 was elevated at 344 (*N* < 35).

Preoperative diagnosis was peritoneal carcinomatosis possibly due to ovarian cancer complicated by pelvic inflammatory disease. The patient underwent a primary debulking procedure including an abdominal hysterectomy, salpingoophorectomy, appendectomy, and omental biopsy. During surgery, bilaterally enlarged ovaries, inflamed fallopian tubes adherent to the pelvic sidewalls, a large amount of ascetic fluid with fibrinoid exudate, and inflammation covering the peritoneal surface, uterus, and bowel were noted. Postoperatively, the patient was treated with ertapenem for peritonitis thought to be secondary to salpingitis; however, fevers, chills, and night sweats persisted.

**Figure 1:**
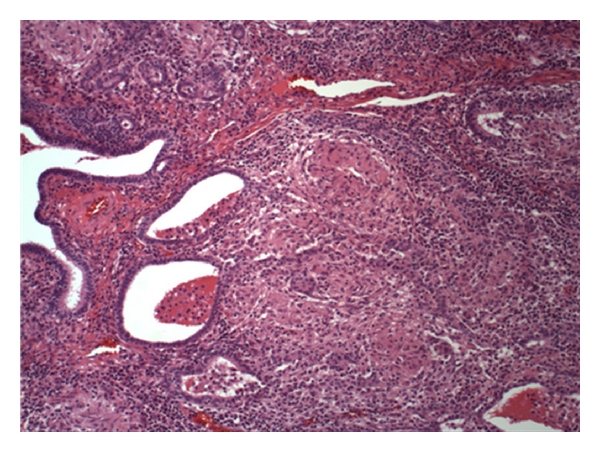
Histopathology showing granulomatous salpingitis.

**Figure 2:**
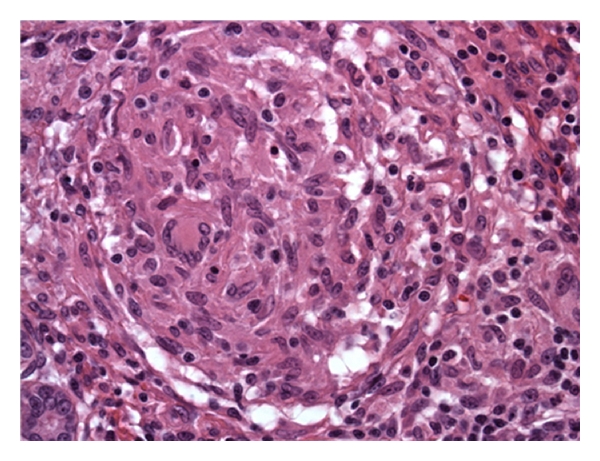
Histopathology showing periovarian caseating granulomatous inflammation.

Histopathology of the resected tissue showed granulomatous salpingitis, periovarian caseating granulomatous inflammation, and granulomatous serositis of the peritoneum (Figures 1 and 2). Subsequently, a T-SPOT.TB (ELISPOT test for interferon gamma levels in the peripheral blood) was positive, and PCR tests of the surgically removed tissues documented the presence of *Mycobacterium tuberculosis*. HIV testing was negative. Thus, the patient was diagnosed with tuberculous peritonitis and started on standard 4-drug antituberculosis treatment. All symptoms resolved. Approximately 6 weeks later, her original ascitic fluid cultures grew *Mycobacterium tuberculosis*.

## 3. Discussion

In this patient, the delay in the diagnosis of tuberculous pertitonitis resulted in what may have been unnecessary radical resection of her reproductive organs. This was unfortunate as peritoneal TB is felt to be medically manageable with the standard four-drug antituberculous regimen for 6 months with the expectation that symptoms will start improving after the first week of therapy [2, 6]. If surgery is indicated, delayed surgery following medical management significantly reduces complications [3]. Review of the literature identified five important considerations that are necessary when faced with a similar presentation: non-United States country of origin, age, CA-125, ascitic fluid analysis, and use of intraoperative frozen sections.

As in any differential, what is common is common. Therefore, it is always prudent to keep in mind age and country of origin when faced with the differential of infectious and malignant etiology [1]. Women with abdominal-pelvic TB are usually between 20 and 40 years of age, younger than women with ovarian tumors [4]. In young woman with elevated CA-125, the low sensitivity and the fact that CA-125 is produced by normal epithelial cells (peritoneum, pleura, and pericardium) when inflamed should prompt consideration of causes other than cancer, especially when serosal fluid is present [5, 6]. Once a definitive diagnosis has been made, CA-125 should instead be utilized to track treatment response, as patients with TB peritonitis have been shown to have rapid declines in CA-125 paralleling clinical response and resolution of ascites after antituberculous treatment [6]. Since ascites is a predominant finding in peritoneal TB, paracentesis can offer further evidence through appropriate fluid analysis. While direct smears of ascitic fluid for acid-fast bacilli are diagnostic in <3% of patients adenosine deaminase activity > 30–32 U/L (sensitivity/specificity > 90%), total protein levels > 25 g/L (sensitivity almost 100%), and serum-ascites albumin gradient < 11 g/L (sensitivity 100%, low specificity) can be used to increase the possibility of diagnosing peritoneal TB [3.6]. Unfortunately, various PCR tests have sensitivity upwards of 95% only when used on smear positive patients (<3% of patients) [6]. Laparoscopy is an ideal method for early diagnosis in peritoneal TB with a sensitivity of 93% and specificity of 98% when macroscopic appearance and combined histological findings are used [3.6]. Thus, intraoperative frozen section would serve as the final checkpoint before proceeding with a cancer-indicated debulking procedure including the permanent removal of reproductive organs.

This case outlines the unfortunate consequences of the misdiagnosis of what probably was an antibiotic responsive illness, resulting in an unnecessarily aggressive surgical procedure.
